# Physical activity and protein-intake strategies to prevent sarcopenia in older people

**DOI:** 10.1093/inthealth/ihae064

**Published:** 2025-04-18

**Authors:** Eunjae Lee, In-Dong Kim, Seung-Taek Lim

**Affiliations:** Institute of Sports & Arts Convergence (ISAC), Inha University, Incheon 22212, Republic of Korea; Waseda Institute for Sport Sciences, Waseda University, Saitama 341-0018, Japan; Seoul Robotics High School, Seoul 06363, Republic of Korea; Waseda Institute for Sport Sciences, Waseda University, Saitama 341-0018, Japan; College of General Education, Kookmin University, Seoul 02707, Republic of Korea

**Keywords:** health promotion, older people, physical activity, protein, sarcopenia

## Abstract

**Background:**

This study aimed to determine the physical activity level and protein intake of older people with sarcopenia and investigate the adequate protein intake of older people in Korea.

**Methods:**

A total of 1215 older people were recruited from the ninth Korea National Health and Nutrition Examination Survey. Participants’ physical activity, handgrip strength, appendicular skeletal muscle mass and food intake were assessed.

**Results:**

A one-way ANOVA revealed that the normal group exhibited significantly higher values for moderate-to-vigorous intensity physical activity (male p=0.035 and female p=0.028), total intake kcal (p<0.001), carbohydrate (p<0.001), proteins (p<0.001) and fats (male p<0.001 and female p=0.005) compared with all other groups. Participants who met the recommended protein intake demonstrated significantly higher muscle mass (OR=2.16) and muscle strength (OR=2.31) compared with those who did not meet the recommended protein intake. A significant positive correlation between protein intake and skeletal muscle index (r=0.354, p<0.001) and handgrip strength (r=0.358, p<0.001) was observed across all participants.

**Conclusion:**

Older individuals who do not meet the recommended protein intake are more likely to experience a loss of muscle mass and strength compared with those who receive the recommended protein intake.

## Introduction

According to statistics, the proportion of people aged ≥65 y in South Korea in 2023 was 18.4% of the total population, a number that is expected to grow, with Korea becoming a super-elderly society.^1^ As the population of older people increases, health becomes a growing societal concern for issues such as sarcopenia. Sarcopenia refers to the loss of muscle mass and strength that accompanies ageing.^2^ The prevalence of sarcopenia increases with age, ranging from 5%–13% at 60–70 y to 11%–50% at >80 y.^3^ Additionally, sarcopenia increases the likelihood of falling and can lead to osteoporosis and obesity, compromising metabolic health.^4^ It was classified by the International Classification of Diseases in 2016 with the code name M62.84.^5^ Moreover, sarcopenia is predicted to affect >500 million older people by 2050.^6^

Proper nutrition and exercise in older adults can prevent sarcopenia. Proper nutrition in older people is critical, as energy intake decreases with age and can decrease by 16%–20%, especially in those aged >65 y.^7^ High-quality protein intake, especially in older adults, can reduce the loss of skeletal muscle mass and strength.^8^ The nutrition programme mainly includes the recommended protein intake of 1.0–1.5 g/kg, of which the proportion of high-quality protein reaches 50%.^9^ However, most of the evidence comes from intervention studies combined with resistance exercise training. Only a few prospective studies have investigated the role of habitual low protein intake in the risk of sarcopenia.^10^

Many studies recommend combining protein intake with physical activity and/or exercise. Compared with low-protein control, protein intake had no significant positive effect on total lean body mass, appendicular skeletal muscle mass (ASM) and handgrip strength, but a statistically significant positive effect was observed on ASM and handgrip strength in the intervention with protein intake and resistance exercise.^11^ Compared with the control group, exercise alone or exercise and protein intake significantly increased handgrip strength and improved dynamic balance, suggesting that both exercise and protein intake combinations have beneficial effects on muscle strength and physical performance in older people with sarcopenia.^12^

Protein intake and physical activity and/or exercise are very important for older people with sarcopenia. However, there was no significant difference in physical activity between exercise intervention and comprehensive intervention groups (exercise + nutrition), which explains why older people did not observe changes in muscle function, because they did not participate in recommended physical activity.^9^ Therefore, this study aimed to determine the physical activity level and protein intake of older people with sarcopenia and investigate the adequate protein intake of older people in Korea.

## Methods

### Participants

Data were collected from the ninth Korea National Health and Nutrition Examination Survey (KNHANES) (2022) conducted by the Ministry of Health and Welfare of Korea.^13^ This study followed the reporting guidelines for cross-sectional research studies outlined in the Strengthening the Reporting of Observational Studies in Epidemiology (STROBE) guidelines.^14^

**Figure 1.**
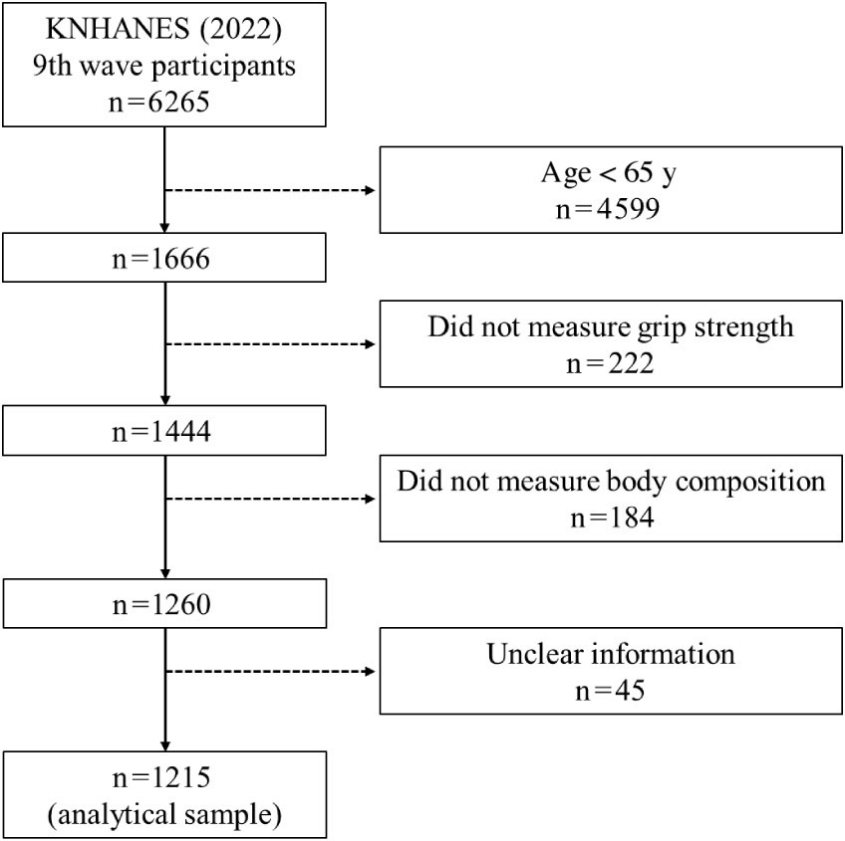
Flowchart detailing study sample selection.

In total, 1215 participants (males=561, females=654) were analysed in this study based on the following criteria: (i) age **≥**65 y; (ii) they had body composition measurements; and (iii) they had handgrip strength measurements. Other participants were excluded and not used in the analysis (Figure 1). Additionally, sarcopenia was determined based on decreased height-adjusted (cm) muscle mass and decreased handgrip strength.^15^

The Institutional Review Board of the Korea Disease Control and Prevention Agency (KDCA) (2018–01–03–4C-A) approved the ninth KNHANES.^16^ In the original survey, informed consent was acquired from all participants. Raw data for this study were sourced through legitimate procedures from the KNHANES website under the KDCA, and all personal data were fully anonymised.

Participant characteristics are provided in Table 1.

**Table 1.** Participant characteristics

	Total (1215)	Male (n=561)	Female (n=654)	p-value
Body composition				
Age (y)	72.38 ± 5.11	72.43 ± 5.13	72.33 ± 5.10	.749
Height (cm)	159.47 ± 8.69	166.71 ± 5.57	153.25 ± 5.48	<.001
Weight (kg)	61.46 ± 10.23	66.53 ± 9.61	57.11 ± 8.63	<.001
BMI (kg/m^2^)	24.12 ± 3.19	23.91 ± 3.00	24.30 ± 3.33	.033
SBP (mmHg)	127.05 ± 16.96	127.00 ± 16.69	127.05 ± 17.21	.934
DBP (mmHg)	73.40 ± 9.08	74.26 ± 9.40	72.66 ± 8.75	.002
Sarcopenia criteria				
SMI (kg/m^2^)	6.65 ± 0.98	7.38 ± 0.74	6.02 ± 0.68	<.001
Right arm (kg)	2.28 ± 0.61	2.77 ± 0.48	1.86 ± 0.33	<.001
Left arm (kg)	2.26 ± 0.60	2.74 ± 0.46	1.85 ± 0.32	<.001
Right leg (kg)	6.32 ± 1.48	7.57 ± 1.04	5.25 ± 0.80	<.001
Left leg (kg)	6.29 ± 1.45	7.51 ± 1.02	5.23 ± 0.80	<.001
Right grip (kg)	26.50 ± 8.23	32.96 ± 6.52	20.73 ± 4.46	<.001
Left grip (kg)	25.33 ± 8.14	31.69 ± 6.52	19.61 ± 4.31	<.001

BMI, body mass index; DBP, diastolic blood pressure; SBP, systolic blood pressure; SMI, skeletal muscle index.

### Diagnosing sarcopenia

Muscle mass and strength were assessed with reference to the algorithms for the community in the Asian Working Group for Sarcopenia 2019 consensus of the sarcopenia criteria.^15^ For skeletal muscle evaluation, we used data measured by the bioelectrical impedance analysis (BIA) method. The skeletal muscle index (SMI) was calculated as the total ASM of the upper arm and lower leg muscles, measured by the BIA method, divided by the square of the height. The low muscle mass diagnostic cut-offs were <7.0 kg/m^2^ for males and <5.7 kg/m^2^ for females. Handgrip strength was used to indicate muscle strength and was measured using a handgrip dynamometer (TKK 5401 Grip-D; Takei, Niigata, Japan). The handgrip low muscle strength diagnostic cut-offs were <28.0 kg for males and <18.0 kg for females.

Based on the aforementioned criteria, we categorised the participants into four groups: those with low muscle mass (ML), those with low muscle strength (SL), those with both low muscle mass and strength (ML+SL) and the normal group.

### Physical activity levels

The current study adopted the computerised Korean version of the International Physical Activity Questionnaire (IPAQ), specifically, the long, self-administered format for a typical week, as outlined in the IPAQ operations manual. The seven-item IPAQ captures the total minutes spent during the preceding 7 d in moderate-to-vigorous intensity physical activity (MVPA), walking and sedentary behaviour.^17^ It is tailored to gather detailed information on the duration (number of sessions and average length per session) of walking, moderate-intensity physical activity (MPA), vigorous-intensity physical activity (VPA) and sedentary behaviour (sitting during weekdays and weekends). The questionnaire includes specific examples to aid participants in reporting moderate and vigorous physical activities. The responses were aggregated for each category (vigorous intensity, moderate intensity and walking) to calculate the total duration of physical activity per week.

### Assessment of carbohydrate, protein and fat intake

A survey was conducted to assess the intake of nutrients, such as dietary carbohydrates, proteins and fats, by visiting specific households according to the KNHANES user guide. Food intake over 24 h was estimated through individual dietary recall interviews. The nutrient intake from food alone, excluding dietary supplements, was calculated. Nutrient intake was defined as the sum of all food and nutrients consumed by one individual during 1 d.

Participants were asked what and how much they ate during the day yesterday to assess nutrient intake. The investigator manually recorded all the foods consumed by the participant the previous day on a food intake survey sheet. Tertiary food codenames were assigned to convert food intake to nutrient intake. The analyses included the following variables: total energy, carbohydrate intake, protein intake and fat intake.

### Statistical analysis

Results are presented as mean±SD. Data analysis was performed using SPSS Statistics for Windows, version 25.0 (IBM Corp., Armonk, NY, USA). The participants’ characteristics (age, height, weight, body mass index, systolic and diastolic blood pressure, ASM and handgrip strength) were further analysed for significant differences between genders using one-way ANOVA. One-way ANOVAs of physical activity and food intake were conducted within four groups. The age-group differences were assessed using a post-hoc Bonferroni test if ANOVA results were significant. Binary logistic regression analyses were performed to investigate independent and joint effects of muscle mass loss and muscle strength loss on protein intake. ORs and 95% CIs were calculated for these relationships. The reference group for the joint association analysis consisted of individuals who met the Dietary Reference Intakes for Koreans (KDRIs)-based protein intake guidelines (60 g of protein intake for males aged ≥65 y and 50 g of protein intake for females aged ≥65 y). Covariates were adjusted in the analyses and included sex (male and female), body mass index (<25 and ≥25 kg/m^2^), smoking status (smoker and non-smoker) and alcohol consumption (drinker and non-drinker). Correlations between protein intake, SMI and handgrip strength were assessed using Pearson's correlation coefficients. The statistical significance level was set at p<0.05.

## Results

### Amount of physical activity and food intake

Tables 2 and 3 present the amount of physical activity and food intake by group.

**Table 2.** Physical activity and food intake by males

	Males (n=534)					
Variable	Normal (n=330)^a^	ML (n=89)^b^	SL (n=57)^c^	ML+SL (n=58)^d^	p-value	Post-hoc
MPA (min/wk)	185.3 ± 165.0	100.8 ± 81.1	110.0 ± 60.8	33.3 ± 16.1	.074	-
VPA (min/wk)	15.6 ± 48.9	7.2 ± 13.7	0.0 ± 0.0	0.0 ± 0.0	.787	-
MVPA (min/wk)	200.9 ± 164.4	108.0 ± 73.9	110.0 ± 60.8	33.3 ± 16.1	.035	-
Total intake of calories (kcal)	2002.2 ± 669.7	1754.3 ± 580.4	1815.7 ± 648.0	1570.2 ± 422.9	<.001	a>b,d
Carbohydrate (g)	318.3 ± 111.7	277.1 ± 89.0	285.4 ± 96.0	261.0 ± 87.2	<.001	a>b,d
Proteins (g)	73.1 ± 28.6	62.5 ± 27.0	66.2 ± 33.9	55.6 ± 20.8	<.001	a>b,d
Fats (g)	41.5 ± 25.2	37.9 ± 24.8	40.3 ± 28.8	30.3 ± 15.9	.015	a>d

ML, low muscle mass; MPA, moderate-intensity physical activity; MVPA, moderate-to-vigorous intensity physical activity; SL, low muscle strength; VPA, vigorous-intensity physical activity.

a normal group.

b ML group.

c SL group.

d ML+SL group.

**Table 3.** Physical activity and food intake by females

	Females (n=591)					
Variable	Normal (n=344)^a^	ML (n=101)^b^	SL (n=73)^c^	ML+SL (n=73)^d^	p-value	Post-hoc
MPA (min/wk)	225.2 ± 195.6	151.3 ± 86.0	26.7 ± 28.9	23.3 ± 15.3	.043	-
VPA (min/wk)	9.5 ± 42.8	2.5 ± 10.0	0.0 ± 0.0	0.0 ± 0.0	.862	-
MVPA (min/wk)	234.7 ± 195.5	153.8 ± 83.7	26.7 ± 28.9	23.3 ± 15.3	.028	-
Total intake of calories (kcal)	1516.1 ± 555.4	1335.9 ± 518.4	1261.8 ± 473.8	1253.4 ± 534.2	<.001	a>b,c,d
Carbohydrate (g)	249.9 ±95.8	216.7 ± 80.6	210.4 ± 74.9	216.9 ± 74.7	<.001	a>b,c,d
Proteins (g)	55.48±24.0	48.31 ± 21.4	46.58 ± 21.5	41.38 ± 14.9	<.001	a > b,c,d
Fats (g)	31.9±20.4	30.43 ± 24.8	25.64 ± 16.5	23.85 ± 14.3	.005	a > d

ML, low muscle mass; MPA, moderate-intensity physical activity; MVPA, moderate-to-vigorous intensity physical activity; SL, low muscle strength; VPA, vigorous-intensity physical activity.

a normal group.

b ML group.

c SL group.

d ML+SL group.

A one-way ANOVA revealed that, among males, the normal group exhibited significantly higher values for MVPA (p=0.035), total intake of calories (p<0.001), carbohydrate intake (p<0.001), protein intake (p<0.001) and fat intake (p<0.001) compared with the ML, SL and ML+SL groups (Table 2). No significant differences were observed in MPA and VPA.

Among females, MVPA (p=0.028), MPA (p=0.043), total intake of calories (p<0.001), carbohydrate intake (p<0.001), protein intake (p<0.001) and fat intake (p=0.005) were significantly higher in the normal group compared with the ML, SL and ML+SL groups (Table 3). No significant differences were observed in VPA.

### Associations between protein intake and muscle mass and strength

Table 4 shows the independent associations between protein intake and muscle mass and strength among older participants.

**Table 4.** Independent associations of protein intake with muscle mass and strength in older people

	Unadjusted		Adjusted^a^	
	OR (95% CI)	p-value	OR (95% CI)	p-value
Loss of muscle, odds Protein intake above the KDRIs Protein intake below KDRIs	1.00 2.16 (1.67–2.80)	<.001	1.00 2.43 (1.83–3.24)	<.001
Loss of strength, odds Protein intake above the KDRIs Protein intake below KDRIs	1.00 2.31 (1.73–3.07)	<.001	1.00 2.19 (1.64–2.92)	<.001

KDRIs, Dietary Reference Intakes for Koreans.

a Adjusted for sex (male and female), body mass index (<25 and ≥25 kg/m^2^), smoking status (smoker and non-smoker) and alcohol consumption (drinkers and non-drinkers).

In the unadjusted model, participants who met the recommended protein intake (OR=2.16, 95% CI 1.67 to 2.80) demonstrated significantly higher levels of muscle mass compared with those who did not meet the recommended protein intake. Those failing to meet the recommended protein intake were 2.16 times more likely to exhibit loss of muscle mass. After adjusting for covariates such as sex, body mass index, smoking status and alcohol consumption, the association between meeting the recommended protein intake and loss of muscle mass was attenuated (OR=2.43, 95% CI 1.83 to 3.24). Nevertheless, even after adjustment, older people who did not meet the recommended protein intake remained 2.43 times more likely to experience loss of muscle mass.

Similarly, in the unadjusted model, participants who met the recommended protein intake (OR=2.31, 95% CI 1.73 to 3.07) were more likely to exhibit higher levels of muscle strength compared with those who did not meet the recommended protein intake. Individuals not meeting the recommended protein intake were 2.31 times more likely to show signs of muscle strength loss. Upon adjusting for covariates (sex, body mass index, smoking status and alcohol consumption), the strength of this association diminished slightly (OR=2.19, 95% CI 1.64 to 2.92). Even with these adjustments, older people who did not meet the recommended protein intake remained 2.19 times more likely to experience loss of muscle strength.

### Correlations coefficients between protein intake with SMI and handgrip strength

**Figure 2.**
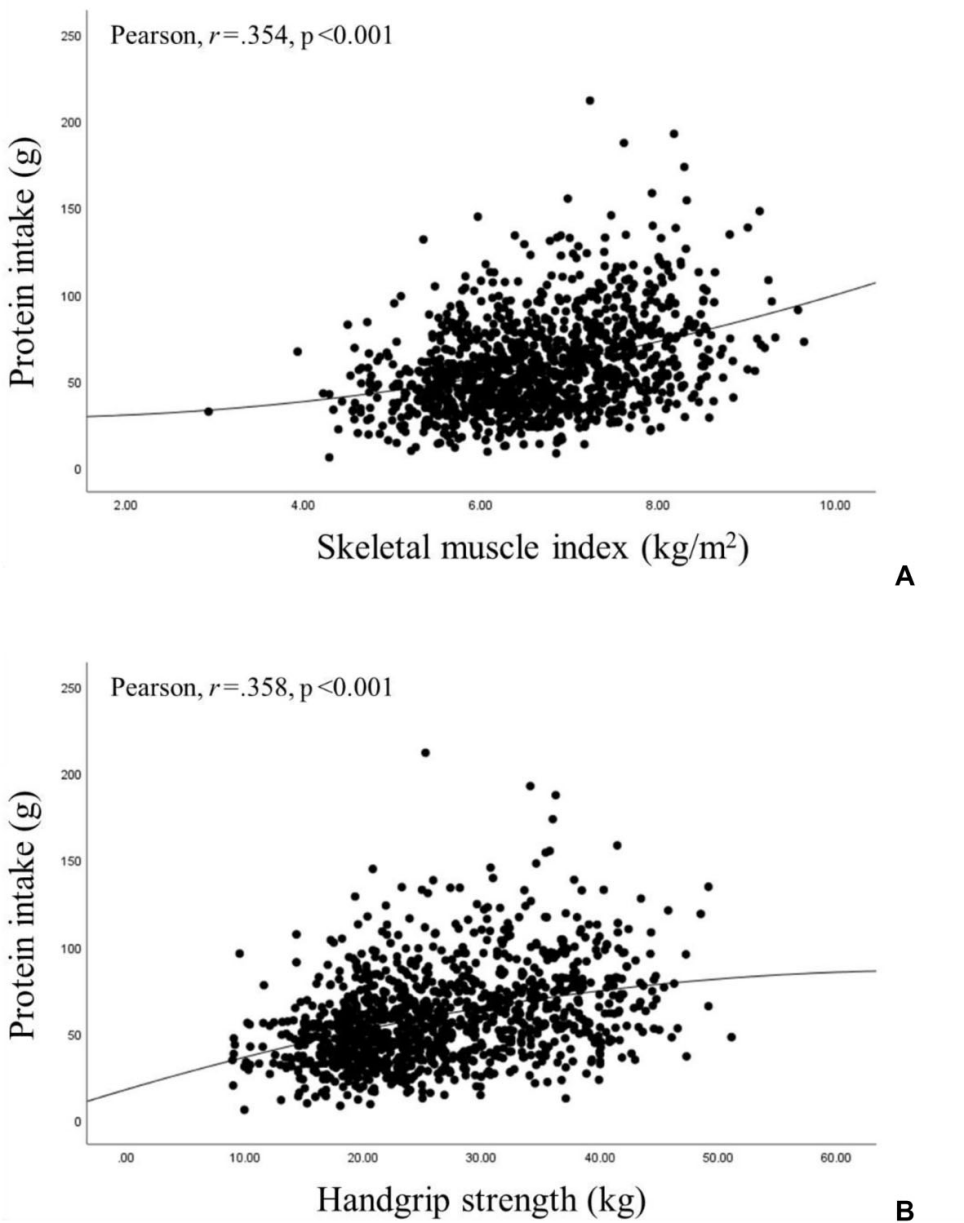
Pearson's correlation coefficients for protein intake with skeletal muscle index and handgrip strength.

Figure 2 shows the correlation coefficients between protein intake with SMI and handgrip strength. A significant positive correlation was observed between protein intake and SMI (r=0.354, p<0.001) (Figure 2A) and between protein intake and handgrip strength (r=0.358, p<0.001) (Figure 2B) across all participants.

## Discussion

This study mainly found that older people who did not meet the recommended protein intake exhibited a decline in muscle mass that was approximately 2.16 times greater compared with those who met the recommended protein intake. Similarly, participants who did not meet the recommended protein intake exhibited a decline in muscle strength that was approximately 2.31 times greater compared with those who met the recommended protein intake. Additionally, increasing protein intake was positively correlated with increasing SMI and handgrip strength.

Muscle is the most essential body component and plays a pivotal role in maintaining a healthy life, being directly or indirectly related to strength, energy balance and/or immunity. Basal metabolic rate decreases during normal ageing, with involuntary muscle loss of 1%–2% per decade after the age of 30 y and approximately 3%–8% per year after the age of 50 y.^18^ Sarcopenia, a progressive and generalised skeletal muscle disorder characterised by accelerated loss of muscle mass and function, is associated with negative outcomes, including falls, functional decline, frailty and mortality.^19^ There are two most effective ways to prevent and improve sarcopenia in older people. First, physical activity and/or exercise have a positive effect on muscle mass and muscle function in people aged >65 y.^20^ Second, physical activity, exercise and adequate protein intake positively impact muscle protein synthesis and, thus, the prevention and treatment of sarcopenia.^21^ While most studies agree on the benefits of physical activity or exercise, the research on protein intake is still controversial. This study examined the physical activity and food intake of South Koreans aged ≥65 y according to their form of sarcopenia. Older males and females shows significant differences in MVPA, and food intake was higher in the normal group than in the sarcopenia groups (ML, SL and ML+SL). Evidence from systematic reviews, meta-analyses and randomised controlled trials showed the effects of exercise training on muscle mass, strength and physical performance in older people with sarcopenia.^22^ Resistance physical activity or exercise, which involves low repetitions against moderate-to-high resistance and contractions of different muscle groups, primarily influences muscle mass and strength in older people with sarcopenia.^23^ Aerobic physical activity or exercise, which involves high repetitions and large muscle groups, increases mitochondrial energy production and capillary density to improve oxygen extraction and muscular endurance.^24^ Resistance training is more commonly recommended for older people with sarcopenia, but both forms of physical exercise possess a common feature: both involve muscle contraction. Therefore, older people with sarcopenia should be actively engaging in muscle contraction activities. Skeletal muscle contraction is associated with the generation of reactive oxygen species, which can act as signalling molecules that stimulate cellular adaptation through the activation of redox-sensitive pathways, including nuclear factor-kB, mitogen‐activated protein kinases and peroxisome proliferator-activated receptor-γ activator-1α.^25^ These signalling pathways mediate some effects of physical exercise, including mitochondrial biogenesis, antioxidant defence, inflammation, apoptosis, autophagy and protein turnover.^26^ Therefore, protein intake in older people with sarcopenia is important to influence protein turnover and cell growth to maintain muscle mass, function and homeostasis.

The availability of amino acids, the building blocks of proteins, is an important factor in regulating muscle protein metabolism, and the interaction between postphysical exercise metabolic processes and increased amino acid availability maximises the stimulation of muscle protein synthesis.^27^ Sarcopenia is a condition in which the body, already depleted of protein due to ageing, cannot withstand protein catabolism resulting from acute illness or inadequate protein intake.^28^ In participants with low protein intake, a longitudinal decline in muscle function (neuromuscular function and strength at 3 wk) was observed before changes in muscle mass (2.3 kg at 9 wk).^29^ Protein intake higher than the recommended dietary allowance is associated with higher short physical performance battery scores, faster walking speed and improved lower extremity and isometric strength and balance.^30^ Our study provides compelling evidence that older individuals who did not meet the recommended protein intake were 2.16 times more likely to experience loss of muscle mass than those who did meet these recommendations. Additionally, those who did not meet the recommended protein intake were 2.31 times more likely to exhibit loss of muscle strength. If protein intake is lower than the body's daily need, then this might cause protein imbalance, skeletal muscle atrophy, impaired muscle growth and decreased function. Thus, consuming enough protein is important to prevent muscle wasting and maintain skeletal muscle mass and function in older people.^31^ The current recommended dietary allowance (RDA) for protein intake (0.8 g protein/kg of body weight/d) is the same for all adults, regardless of age or gender.^32^ The estimated average requirement (EAR) is the average daily nutrient intake level (0.66 g/kg of body weight/d) necessary to meet the protein needs of one-half of healthy adults.^33^ Both the RDA and EAR are Western standards, and the recommended protein intake for Koreans is 60 g/d for males and 50 g/d for females.^34^ Discussions and research on protein intake are ongoing, with many differing opinions based on demographics, health conditions and other factors. The RDA protein recommendation for older people is low; for optimal health, older adults should consume 1.0–1.2 g/kg body weight/d.^35^ In this study, we found a positive association between protein intake and SMI and handgrip strength in older people. These findings are supported by evidence that while it might be necessary to increase protein intake, meeting a minimum protein intake recommendation is very important.

The current study has some limitations that warrant attention. First, the current data from the ninth KNHANES are only available for ASM measured by the BIA method. In the future, analysing more precise data by measuring ASM through dual-energy X-ray absorptiometry will be necessary. Second, although the study targeted adults aged ≥65 y, the medical conditions of participants were not assessed. Finally, because the evaluation of physical activity was conducted by a questionnaire, an objective evaluation should be made in future studies. In addition to physical activity, resistance training is a popular way to increase muscle mass. Further intervention studies of protein intake and resistance training in older people with sarcopenia are needed.

### Conclusion

This study demonstrates the relationship between protein intake and muscle mass and strength among older people. Our findings offer robust evidence that older individuals who do not meet the recommended protein intake are more likely to experience a loss of muscle mass and strength compared with those who meet the recommended protein intake. Additionally, we found that MVPA was significantly higher in the normal group compared with all other groups. In older individuals, the OR was even more pronounced, underscoring the importance of increased MVPA and protein intake. Therefore, we strongly advocate for increasing protein intake and physical activity to mitigate sarcopenia and improve daily living activities for older individuals.

## Data Availability

The data that support the findings of this study are available on request from the corresponding author.

## References

[1] Statistics Korea . 2023 Elderly statistics. 2023.

[2] Rosenberg H . Sarcopenia: origins and clinical relevance. J Nutr. 1997;127:990–1. 10.1093/jn/127.5.990S 9164280

[3] Morley JE . Sarcopenia: diagnosis and treatment. J Nutr Health Aging. 2008;12:452–6.18615226 10.1007/BF02982705

[4] Hunter GR, Singh H, Carter SJ, et al. Sarcopenia and its implications for metabolic health. J Obes. 2019;2019:8031705. 30956817 10.1155/2019/8031705 PMC6431367

[5] Cao L, Morley JE. Sarcopenia is recognized as an independent condition by an international classification of disease, tenth revision, clinical modification (ICD-10-CM) code. J Am Med Dir Assoc. 2016;17:675–7. 27470918 10.1016/j.jamda.2016.06.001

[6] Cruz-Jentoft AJ, Baeyens JP, Bauer JM, et al. Sarcopenia: European Consensus on definition and diagnosis: Report of the European Working Group on Sarcopenia in older people. Age Ageing. 2010;39:412–23. 20392703 10.1093/ageing/afq034 PMC2886201

[7] Giezenaar C, Chapman I, Luscombe-Marsh N, et al. Ageing is associated with decreases in appetite and energy intake—A meta-analysis in healthy adults. Nutrients. 2016;8:28. 26751475 10.3390/nu8010028 PMC4728642

[8] Scotto DPA, McSwiney FT, Hone M, et al. Effects of a long chain n-3 polyunsaturated fatty acid-rich multi-ingredient nutrition supplement on body composition and physical function in older adults with low skeletal muscle mass. J Diet. 2021;Suppl 1:1–16. 10.1080/19390211.2021.1897057 33759678

[9] Wang Z, Xu X, Gao S, et al. Effects of internet-based nutrition and exercise interventions on the prevention and treatment of sarcopenia in the elderly. Nutrients. 2022;14:2458. 35745187 10.3390/nu14122458 PMC9229368

[10] Robinson S, Granic A, Cruz-Jentoft AJ, et al. The role of nutrition in the prevention of sarcopenia. Am J Clin Nutr. 2023;118:852–64. 37657521 10.1016/j.ajcnut.2023.08.015 PMC10636259

[11] Kirwan RP, Mazidi M, García CR, et al. Protein interventions augment the effect of resistance exercise on appendicular lean mass and handgrip strength in older adults: A systematic review and meta-analysis of randomized controlled trials. Am J Clin Nutr. 2022;115:897–913. 34673936 10.1093/ajcn/nqab355

[12] Wu PY, Huang KS, Chen KM, et al. Exercise, nutrition, and combined Exercise and nutrition in older adults with sarcopenia: A systematic review and network meta-analysis. Maturitas. 2021;145:38–48. 33541561 10.1016/j.maturitas.2020.12.009

[13] The Korea National Health and Nutrition Examination Survey (KNHANES), 2022 . Korea Disease Control and Prevention Agency. 2022.

[14] STROBE. STROBE Checklist: Cross-Sectional Studies . Available at: https://www.strobe-statement.org/ [accessed March 13, 2024].

[15] Chen LK, Woo J, Assantachai P, et al. Asian Working Group for Sarcopenia: 2019 consensus update on Sarcopenia diagnosis and treatment. J Am Med Dir Assoc. 2020;21:300–7. 32033882 10.1016/j.jamda.2019.12.012

[16] Korea Centers for Disease Control and Prevention Agency . The 9th Korea National Health and Nutrition Examination Survey. Available at: https://knhanes.kdca.go.kr/knhanes/sub03/sub03_02_05.do [accessed March 13, 2024].

[17] Lim ST, Jung YZ, Akama T, et al. Physical activity amount and cognitive impairment in Korean elderly population. Brain Sci. 2020;10:804. 33142716 10.3390/brainsci10110804 PMC7693022

[18] Lim ST, Kang S. Exercise therapy for sarcopenia and diabetes. World J Diabetes. 2023;14:565–72. 37273255 10.4239/wjd.v14.i5.565 PMC10237001

[19] Cruz-Jentoft AJ, Sayer AA. Sarcopenia. Lancet. 2019;393:2636–46. 31171417 10.1016/S0140-6736(19)31138-9

[20] Beaudart C, Dawson A, Shaw SC, et al. Nutrition and physical activity in the prevention and treatment of sarcopenia: Systematic review. Osteoporos Int. 2017;28:1817–33. 28251287 10.1007/s00198-017-3980-9 PMC5457808

[21] Naseeb MA, Volpe SL. Protein and exercise in the prevention of sarcopenia and aging. Nutr Res. 2017;40:1–20. 28473056 10.1016/j.nutres.2017.01.001

[22] Lu L, Mao L, Feng Y, et al. Effects of different exercise training modes on muscle strength and physical performance in older people with sarcopenia: A systematic review and meta-analysis. BMC Geriatr. 2021;21:708. 34911483 10.1186/s12877-021-02642-8 PMC8672633

[23] Vikberg S, Sörlén N, Brandén L, et al. Effects of resistance training on functional strength and muscle mass in 70-year-old individuals with pre-sarcopenia: A randomized controlled trial. J Am Med Dir Assoc. 2019;20:28–34. 30414822 10.1016/j.jamda.2018.09.011

[24] Coelho-Júnior HJ, Calvani R, et al. Engagement in aerobic exercise is associated with a reduced prevalence of sarcopenia and severe sarcopenia in Italian older adults. J Pers Med. 2023;13:655. 37109041 10.3390/jpm13040655 PMC10144721

[25] Marzetti E, Calvani R, Tosato M, et al. Physical activity and exercise as countermeasures to physical frailty and sarcopenia. Aging Clin Exp Res. 2017;29:35–42. 10.1007/s40520-016-0705-4 28181204

[26] Pérez-Baos S, Prieto-Potin I, Román-Blas JA, et al. Mediators and patterns of muscle loss in chronic systemic inflammation. Front Physiol. 2018;9:409. 29740336 10.3389/fphys.2018.00409 PMC5928215

[27] Tipton KD, Wolfe RR. Exercise, protein metabolism, and muscle growth. Int J Sport Nutr Exerc Metab. 2001;11:109–32. 11255140 10.1123/ijsnem.11.1.109

[28] Cruz-Jentoft AJ, Bahat G, Bauer J, et al. Sarcopenia: Revised European consensus on definition and diagnosis. Age Ageing. 2019;48:16–31. 30312372 10.1093/ageing/afy169 PMC6322506

[29] Groot LC, Staveren WA. Low-protein intakes and protein turnover in elderly women. Nutr Rev. 1996;54:58–60. 9053825 10.1111/j.1753-4887.1996.tb03857.x

[30] Coelho-Júnior HJ, Calvani R, Tosato M, et al. Protein intake and physical function in older adults: A systematic review and meta-analysis. Ageing Res Rev. 2022;81:101731. 36087703 10.1016/j.arr.2022.101731

[31] Deer RR, Volpi E. Protein intake and muscle function in older adults. Curr Opin Clin Nutr Metab Care. 2015;18:248–53. 25807346 10.1097/MCO.0000000000000162 PMC4394186

[32] Institute of Medicine . Dietary Reference Intakes for Energy, Carbohydrate, fiber, Fat, Fatty Acids, Cholesterol, Protein and Amino Acids. Washington (DC): National Academies Press; 2005.

[33] Traylor DA, Gorissen SHM, Phillips SM. Perspective: Protein requirements and optimal intakes in aging: Are we ready to recommend more than the recommended daily allowance? Adv Nutr. 2018;9:171–82. 29635313 10.1093/advances/nmy003 PMC5952928

[34] Ministry of Health and Welfare, The Korean Nutrition Society . Application of dietary reference intakes for Koreans 2020. Sejong. 2021.

[35] Paddon-Jones D, Leidy H. Dietary protein and muscle in older persons. Curr Opin Clin Nutr Metab Care. 2014;17:5–11. 24310053 10.1097/MCO.0000000000000011 PMC4162481

